# Tumor Volume Dynamics as an Early Biomarker for Patient-Specific Evolution of Resistance and Progression in Recurrent High-Grade Glioma

**DOI:** 10.3390/jcm9072019

**Published:** 2020-06-27

**Authors:** Daniel J. Glazar, G. Daniel Grass, John A. Arrington, Peter A. Forsyth, Natarajan Raghunand, Hsiang-Hsuan Michael Yu, Solmaz Sahebjam, Heiko Enderling

**Affiliations:** 1 Department of Integrated Mathematical Oncology, H. Lee Moffitt Cancer Center & Research Institute, Tampa, FL 33612, USA; daniel.glazar@moffitt.org; 2 Department of Radiation Oncology, H. Lee Moffitt Cancer Center & Research Institute, Tampa, FL 33612, USA; daniel.grass@moffitt.org (G.D.G.); michael.yu@moffitt.org (H.-H.M.Y.); 3 Department of Oncologic Sciences, University of South Florida, Tampa, FL 33612, USA; john.arrington@moffitt.org (J.A.A.); peter.forsyth@moffitt.org (P.A.F.); natarajan.raghunand@moffitt.org (N.R.); 4 Department of Radiology, H. Lee Moffitt Cancer Center & Research Institute, Tampa, FL 33612, USA; 5 Department of Orthopaedics & Sports Medicine, University of South Florida, Tampa, FL 33612, USA; 6 Department of Radiology, University of South Florida, Tampa, FL 33612, USA; 7 Department of Neuro-Oncology, H. Lee Moffitt Cancer Center & Research Institute, Tampa, FL 33612, USA; 8 Department of Cancer Physiology, H. Lee Moffitt Cancer Center & Research Institute, Tampa, FL 33612, USA

**Keywords:** mathematical model, response prediction, high-grade glioma, patient-specific

## Abstract

Recurrent high-grade glioma (HGG) remains incurable with inevitable evolution of resistance and high inter-patient heterogeneity in time to progression (TTP). Here, we evaluate if early tumor volume response dynamics can calibrate a mathematical model to predict patient-specific resistance to develop opportunities for treatment adaptation for patients with a high risk of progression. A total of 95 T1-weighted contrast-enhanced (T1post) MRIs from 14 patients treated in a phase I clinical trial with hypo-fractionated stereotactic radiation (HFSRT; 6 Gy × 5) plus pembrolizumab (100 or 200 mg, every 3 weeks) and bevacizumab (10 mg/kg, every 2 weeks; NCT02313272) were delineated to derive longitudinal tumor volumes. We developed, calibrated, and validated a mathematical model that simulates and forecasts tumor volume dynamics with rate of resistance evolution as the single patient-specific parameter. Model prediction performance is evaluated based on how early progression is predicted and the number of false-negative predictions. The model with one patient-specific parameter describing the rate of evolution of resistance to therapy fits untrained data ($R^{2}=0.70$). In a leave-one-out study, for the nine patients that had T1post tumor volumes ≥1 cm^3^, the model was able to predict progression on average two imaging cycles early, with a median of 9.3 (range: 3–39.3) weeks early (median progression-free survival was 27.4 weeks). Our results demonstrate that early tumor volume dynamics measured on T1post MRI has the potential to predict progression following the protocol therapy in select patients with recurrent HGG. Future work will include testing on an independent patient dataset and evaluation of the developed framework on T2/FLAIR-derived data.

## 1. Introduction

Gliomas are cancers of glial cells in the brain. High-grade gliomas (HGGs) are particularly aggressive with dismal overall survival. Part of the challenge in treating HGGs is the naturally immune-privileged environment of the brain [1]. Inhibitory proteins, programmed death protein 1 (PD-1), and its ligands (PD-L1 and PD-L2), are expressed in the microenvironment of most HGGs [2,3,4]. Recently, anti-angiogenic therapy, such as bevacizumab, to target vascular endothelial growth factor (VEGF), and immunotherapy, such as pembrolizumab, to target programmed death protein 1 (PD-1) on T lymphocytes have been prospectively evaluated for HGGs [5,6,7,8].

In our clinical trial, patients with recurrent HGG were treated with hypo-fractionated stereotactic radiotherapy (HFSRT) combined with pembrolizumab and bevacizumab (NCT02313272) [9]. Adult patients with recurrent glioblastoma (GBM) or anaplastic astrocytoma (maximum diameter of target lesion ≤3.5 cm) were eligible. Eligible patients received HFSRT to the recurrent tumor (6 Gy × 5) combined with pembrolizumab (100 or 200 mg, intravenously based on dose level, every 3 weeks) and bevacizumab (10 mg/kg, intravenously every 2 weeks). After determination of the recommended phase II dose of pembrolizumab, an additional 26 patients were enrolled in an expansion cohort and were treated with HFSRT, pembrolizumab (200 mg every 3 weeks), and bevacizumab. Response was assessed every 6 weeks per Response Assessment in Neuro-Oncology (RANO) criteria.

The RANO criteria define radiographic progression as 25% or greater increase in the sum of the products of perpendicular diameters of the enhancing lesion, when compared with baseline or smallest tumor measurement (nadir). Additionally, progression may be observed by a significant increase in a T2-weighted/fluid-attenuated inversion recovery (T2/FLAIR) non-enhancing lesion on stable or increasing doses of corticosteroids compared with nadir [10]. The present RANO criteria for HGGs do not include volumetric assessment. While contrast-enhancing volumetric changes were found to be predictive of progression-free survival (PFS), volumetric measures have not yet been incorporated into the RANO criteria [11,12,13].

Despite the advances of drug development, recurrent HGGs remain incurable as patients inevitably develop resistance and progress on treatment. However, time to progression varies significantly between patients, and no reliable biomarkers exist to predict when resistance will develop. Prediction of time to progression requires temporally resolved biomarkers and identification of highly patient-specific therapy response dynamics.

Here, we evaluate if early tumor volume dynamics in T1-weighted contrast-enhanced (T1post) measurements can be used to calibrate and validate a mathematical model of patient-specific tumor volume dynamics to predict response dynamics following therapy. Predicting progression prior to radiographic manifestation allows clinicians to modify therapy before selection for and proliferation of treatment-resistant tumor subpopulations. We identify a subset of recurrent HGG patients for whom treatment response dynamics can accurately predict progression.

## 2. Experimental Section

**Figure 1:**
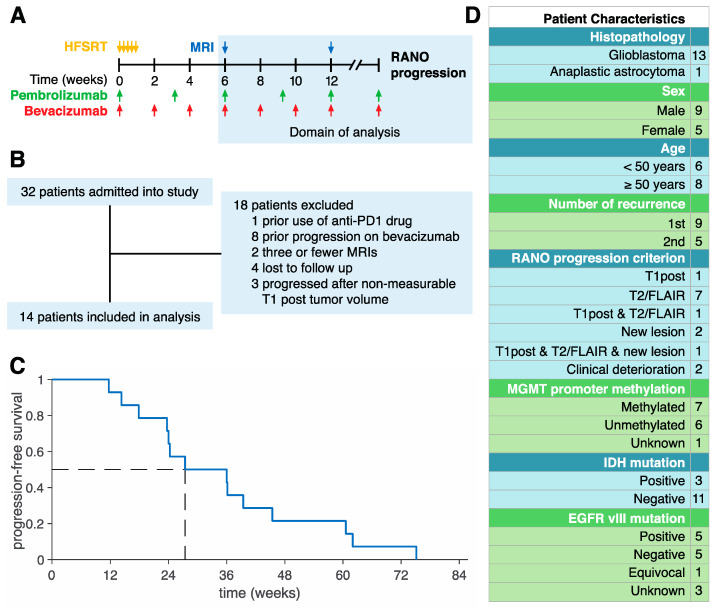
Patient Cohort. (**A**) Schematic of the NCT02313272 protocol. Patients were treated with hypo-fractionated stereotactic radiotherapy (HFSRT; 6 Gy × 5) plus pembrolizumab (100 or 200 mg, every 3 weeks) and bevacizumab (10 mg/kg, every 2 weeks). MRIs were taken approximately every 6 weeks. The shaded region indicates the time domain of the present analysis. (**B**) Exclusion criteria. A total of 32 patients were enrolled into the clinical trial with 14 patients included in the present analysis. (**C**) Progression-free survival for the 14 patients included in the present analysis. The median time to progression was 27.4 weeks. (**D**) Characteristics of 14 patients included in the study.

A total of 32 patients with recurrent HGG were treated with HFSRT (6 Gy × 5) plus pembrolizumab (100 or 200 mg, intravenously based on dose level, every 3 weeks) and bevacizumab (10 mg/kg, intravenously every 2 weeks) until progression in a phase I clinical trial (NCT02313272) at Moffitt Cancer Center between August 2015 and March 2018. T1post and T2-weighted/fluid-attenuated inversion recovery (T2/FLAIR) MRIs were taken approximately every 6 weeks (Figure 1A). The protocol and its amendments were reviewed and approved by the institutional review board (IRB study #: Pro00014674, continuing review approval IRB# 00000971, 26 August 2019). Written consent was provided by all patients. Included in the present analysis were pembrolizumab-naïve and bevacizumab-naïve patients with at least four MRI-derived T1post volumes and who progressed with non-zero T1post volume (Figure 1B). The median time to progression for these patients was 27.4 weeks (Figure 1C).

Of the 14 patients included in the present analysis, three progressed due to a greater than 25% increase in the sum of the products of perpendicular diameters of T1post enhancing lesions compared with nadir, nine progressed due to significant increase in T2/FLAIR non-enhancing lesions, three progressed due to the development of a new lesion, and two progressed due to clinical deterioration (Figure 1D). Notice that two of our patients developed progression under multiple criteria.

Patient-specific longitudinal gross tumor volumes were delineated from a total of 95 T1post MRI data sets (median of 5.5 images per patient, median of 42 days between images). Briefly, for a given patient, consecutive MRI scans were evaluated, and T1post volumes were manually delineated on each slice with commercially available software (Mirada Medical, Denver, CO, USA) by an experienced radiation oncologist (GDG) and were verified by a neuro-radiologist (JAA). For patients who underwent surgery for recurrent disease prior to HFSRT, the volume only included the residual T1post volume excluding the surgical cavity. For patients who did not undergo salvage surgery prior to HFSRT, the T1post volume was delineated from the epicenter of the recurrence. For a given patient, recurrent T1post volumes were concordant in the region of delineation across MRI scans, except when the recurrence was multi-focal. T1post volumes were delineated if they were measurable (i.e., if they were ≥0.1 cm^3^). Non-measurable T1post volumes were set to 0.0 cm^3^.

To evaluate T1post MRI tumor volume dynamics as an early biomarker for progression in recurrent HGG, we followed the rigorous Brady pipeline to develop, calibrate, and validate a mathematical model and evaluate model prediction performance [14]. Simulation of the evolution of tumor volume during the course of therapy traditionally involves the development of a mathematical model that describes the mechanism of each therapeutic agent. This requires numerous parameters that will be difficult to learn from the limited number of patients in the analysis (n = 14) and sparse longitudinal data (median, 5.5 MRIs per patient).

We deployed a simple tumor growth inhibition (TGI) model [15] to describe tumor volume dynamics V(t):

(1) $$\frac{dV}{dt}=\lambda V-\gamma V,$$

where  λ (day^−1^) is the net tumor growth rate in the absence of therapy and γ(t) (day^−1^) is the rate at which the tumor volume decays in response to therapy. We assume an exponential growth as the tumor volume is likely far from carrying capacity after surgery and HFSRT, supported by observed dynamics.

Bevacizumab and pembrolizumab were administered every 2 and 3 weeks until progression; thus, we approximated a continuous and constant drug concentration and ignored pharmacokinetics and pharmacodynamics for simplicity. To simulate the evolution of resistance to therapy, we modeled the decay rate to be exponentially declining with time, such that

(2) $$\frac{d\gamma }{dt}=-\varepsilon \gamma ,$$

where *ε* (day^−1^) is the rate at which resistance develops.

We express the analytic solution of the coupled system of Equations (1) and (2) as:

$$\begin{array}{l} V\left(t\right)=V_{0}\cdot \exp\left[\lambda \cdot \left(t-t_{0}\right)+\frac{1}{\varepsilon }\left(\gamma \left(t\right)-\gamma_{0}\right)\right],\\ \gamma \left(t\right)=\gamma_{0}\cdot \exp\left[-\varepsilon \cdot \left(t-t_{0}\right)\right], \end{array}$$

with initial conditions $\left[V_{0},\gamma_{0}\right]$ evaluated at $t=t_{0}$. Note that time to tumor growth (TTG), defined as time of volume nadir and resistance to therapy, is $\;t=t^{*}$, such that $\lambda =\gamma \left(t^{*}\right)$. Analytically, this occurs at $t^{*}=\frac{\ln\left(\gamma_{0}\right)-\ln\left(\lambda \right)}{\varepsilon }$. A summary of model parameters is given in Table 1. The parameter bounds are defined from the patient dataset.

**Figure 2:**
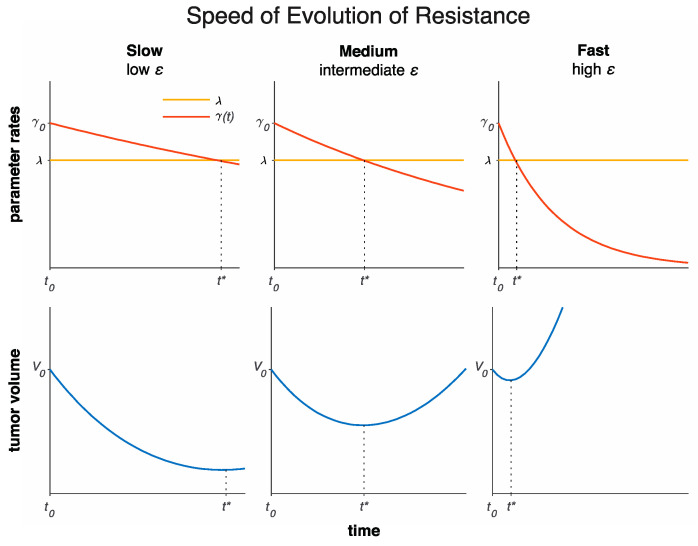
The model can explain a variety of tumor volume dynamics by varying the speed of evolution of resistance to therapy. A low resistance rate ε (left) yields slow evolution of resistance to therapy and long-term response with large tumor volume regression. A medium ε (middle) yields medium evolution of resistance to therapy and short-term response with medium tumor volume regression. A high ε (right) yields fast evolution of resistance to therapy and immediate progression with small tumor volume regression. Resistance to therapy and volume nadir occur at $t=t^{*}$, such that the net growth rate and treatment sensitivity coincide ($\lambda =\gamma \left(t^{*}\right)$ ). The legend in top left panel applies to all top panels.

To make predictions over sparse data, we need to minimize the number of free parameters. Patient-specific tumor proliferation and invasion have been thoroughly investigated in mathematical models of glioma [16,17,18,19,20,21,22,23,24]. Here, sensitivity analysis of Equations (1) and (2) (Supplementary Information, [25]), identified the rate of evolution of resistance as the most sensitive parameter. Therefore, we modeled *ε* to be patient-specific and set less-sensitive parameters (net tumor growth rate λ and treatment response rate $\gamma_{0}$) to be uniform across the patient cohort (Table 1). This model has the advantage of being very simple (only one parameter to be trained per patient), as well as being able to explain a variety of tumor volume dynamics (Figure 2).

We also performed an identifiability analysis, detailed in the Supplementary Information [26,27]. In case of non-identifiability, we fixed the least sensitive uniform parameter to some nominal value. Model parameters are derived by fitting the analytic solution of the mathematical model to the clinical data by minimizing the sum of squared relative errors $E=\frac{V_{\mathrm{sim}}-V_{\mathrm{data}}}{\left(V_{\mathrm{sim}}+V_{\mathrm{data}}\right)/2}$ [28], where $V_{\mathrm{sim}}$ is simulated volume at the time of MRI, and $V_{\mathrm{data}}$ is the actual volume at the time of MRI, over all MRIs. This relative error was chosen to avoid fitting artefacts to non-measurable tumor volumes. To best fit to resistance dynamics, the model solution was fixed to the final measurable tumor volume and simulated back in time, giving double weight to the penultimate tumor volume. Optimal parameters are derived by implementing a nested particle swarm algorithm in MATLAB R2020a.

To validate the calibrated model with untrained data, we performed a leave-one-out cross-validation (LOOCV) study. We calibrated the uniform model parameters using the training dataset and applied them to the left-out patient to learn the patient-specific parameter. We performed this for all patients and aggregate results. Model calibration and validation were evaluated based on coefficient of determination (R^2^).

**Figure 3:**
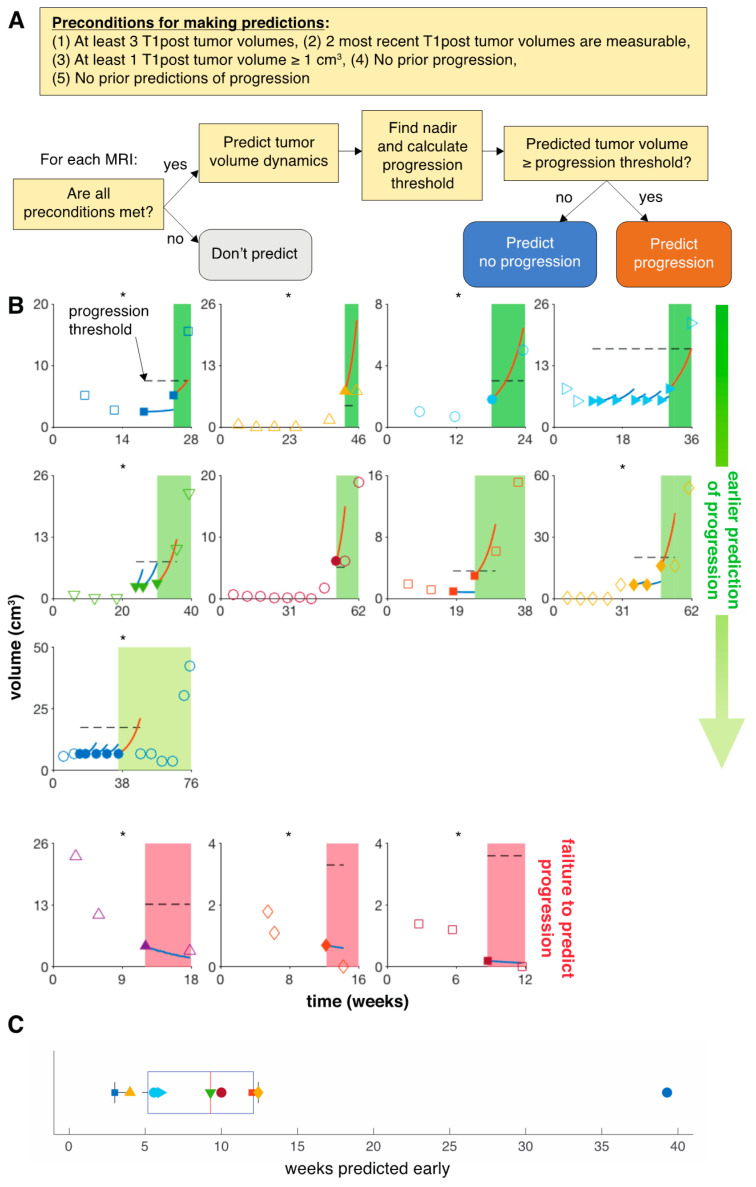
Model predicts progression for 9 of 14 patients with recurrent HGG in a LOOCV. (**A**) Decision diagram for making predictions. (**B**) Using the 200% increase in T1post tumor volume from nadir progression criterion, which maximizes NPV, we follow the decision diagram for each MRI. No predictions were made for 2 patients (not shown) due to no T1post tumor volume ≥1 cm^3^. Of the 9 patients predicted to progress, four were predicted to progress one scan early, four were predicted to progress two scans early, and one was predicted to progress six scans early. Filled markers identify the radiological data from which the model forecast has been performed. Blue and red curves show the model prediction relative to the progression threshold (dashed line). Asterisks mark patients who progressed due to T2/FLAIR non-enhancing lesions or clinical deterioration. All three patients whose progression failed to be predicted progressed despite accurately predicted diminished T1post volumes. Two of these patients progressed due to significant increase in T2/FLAIR non-enhancing lesions, and one progressed due to clinical deterioration. (**C**) Distribution of early predictions. Markers correspond to individual patients in panel (B).

We then predicted tumor volume and response dynamics for each individual patient. We decided to make predictions only when the following conditions were met: 1. There are at least three T1post tumor volumes while on the present treatment; 2. The two most recent T1post tumor volumes are measurable; 3. At least one T1post tumor volume is greater than 1 cm^3^, which is a three-dimensional approximation of the 1 cm longest diameter criteria in RANO; 4. There is no prior progression; and 5. There is no prior prediction of progression (Figure 3A). Once those conditions are met, we set the initial condition to the most recent tumor volume and back fit to the first data point, learning the patient-specific resistance rate ε (day^−1^) using the uniform parameters learned from the training cohort. Tumor volume was then predicted for the next time point. Progression or no progression is evaluated based on the predicted tumor volume relative to nadir. A variety of thresholds relative to nadir were tested (0–300% increase in tumor volume from nadir) to determine the optimal progression criterion. The threshold that maximizes the negative predictive value (NPV) was selected as optimal for predicting response dynamics. Model predictive performance was evaluated based on minimizing false-negative (FN) predictions, as well as minimizing the number of weeks for which progression is predicted early for each patient. Model calibration, validation, and predictions were performed in 20 replicates to average numerical stochasticity. Statistics are reported as mean and standard deviation unless otherwise stated.

## 3. Results

### 3.1. Model Fits to Patient Data

**Figure 4:**
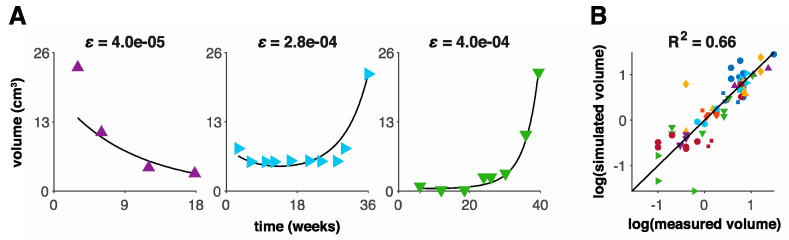
Model validation. (**A**) Fits of three representative patients (low, medium, and high ε). (**B**) Model fits to the patient dataset with $R^{2}=0.66$ for one particular leave-one-out cross-validation (LOOCV) replicate. Each point represents a single MRI scan, and each symbol represents a single patient. The calculation of the coefficient of determination (R^2^) was based on absolute tumor volumes without logarithmic transformation.

The tumor volume growth and decay model exhibits a variety of dynamics dependent on the resistance rate ε (Figure 2). A low ε yields slow evolution of resistance and long-term response and volume regression. A high ε yields fast evolution of resistance and short-term response or even immediate progression. The model with three patient-specific parameters (net growth rate *λ* initial treatment sensitivity $\gamma_{0}$, and resistance rate  ε) fits the volumetric data of the analyzed 14 patients with recurrent HGG treated with HFSRT with concurrent pembrolizumab and bevacizumab with median $R^{2}=0.76$. The resistance rate ε was identified as the most sensitive parameter; thus, we kept ε to be patient-specific, and we trained λ and $\gamma_{0}$ to be uniform across all patients (Figure S1). While this significantly simplifies the model, it remains practically non-identifiable. Because $\gamma_{0}$ was found to be the least sensitive parameter, we set it to the nominal value $\gamma_{0}=0.4608$ day^−1^, which maximizes R^2^ and optimized for λ=0.4465±0.0023  day^−1^ (Figure S2). Model validation via the LOOCV study was performed with median $R^{2}=0.66$ on this patient dataset (Figure 4).

### 3.2. Model Predicts Early Progression in 9 of 14 Patients

We evaluated a variety of T1post volumetric progression criteria and selected the one that maximized the negative predictive value (NPV). NPV minimized predicting no progression when there was in fact progression (false negative, FN) and maximized predicting no progression correctly to minimize the number of MRI scans that we predicted early (true negative, TN). Of all the progression criteria sampled, a 200% increase in tumor volume from nadir maximizes the NPV at 0.84 with a sensitivity of 0.56 and a specificity of 0.76 (Table 2).

The chosen 200% increase in tumor volume from nadir also minimizes the time that progression is predicted early. In line with RANO, no predictions were made for two patients because of no T1post tumor volumes ≥1 cm^3^ prior to progression. Our model predicts progression early in 9 of the remaining 12 patients. Of these, four were correctly predicted to progress one scan early, four were predicted to progress two scans in advance, and one patient was predicted to progress six scans early (Figure 3B). Progression was predicted with a median of 9.3 (range: 3–39.3) weeks early (Figure 3C), despite the fact that 6 of these 9 patients (66%) progressed due to significant increase in T2/FLAIR non-enhancing lesions or due to clinical deterioration.

For three patients, the model accurately forecasted the evolution of the target lesion but failed to predict progression early due to significant increase in T2/FLAIR non-enhancing lesions or clinical deterioration (Figure 3B).

## 4. Discussion

We have developed a simple mathematical model to simulate T1post tumor volume dynamics during therapy as a predictor for progression to subsequent therapy. As temporally resolved volumetric data are only collected every 6 weeks, the model has to be very simple, and to our knowledge, this is the first attempt to simulate treatment resistance as a patient-specific exponential decline of treatment sensitivity. Using this model, we identified recurrent HGG patients who were treated with HFSRT combined with pembrolizumab and bevacizumab without previous drug failure and with enhancing tumor volume greater than 1 cm^3^, for whom predictions can be made with high confidence.

To this extent, we have developed a novel volumetric progression classifier of 200% above nadir. However, predicted tumor volumes near the progression threshold can potentially skew the results. This may be mediated by assigning prediction confidence based on how far the predicted tumor volume is away from the progression threshold. Additionally, this figure seems surprisingly high, given that the equivalent volumetric threshold to the current RANO bidirectional criterion assuming a spherical tumor is 40%, warranting further investigations into tumor volume dynamics. However, tumor contouring was done by a radiologist or radiation oncologist and remains a manual process as the ability of auto-contouring the tumor on T1post images is not yet fully commercially available. Current software allows auto-contours of normal structures to a high-degree precision but may still require manual adjustment.

The developed model may provide medical oncologists with an additional tool to discuss treatment options on a per patient basis. One limitation of the presented study is the limited patient dataset (n = 14 patients with 95 MRIs in total). The inclusion of more patients would further refine model calibration and progression criterion selection, with the hope to identify different progression models based on additional patient biomarkers, such as lymphocyte and neutrophil dynamics, MGMT promoter methylation, IDH mutation, and EGFR vIII mutation.

Our results demonstrate that early tumor volume dynamics in T1post measurements have the potential to predict progression. However, using T1post volumetric measurements to predict progression has limitations with therapies that include anti-angiogenic agents, which have a propensity to exhibit pseudo-response in T1post MRIs. This is illustrated by 3 of 14 patients in the discussed cohort with shrinking T1post tumor volumes and thus predicted to continue therapy without disease progression, yet who progressed due to significant increase in T2/FLAIR non-enhancing lesions or clinical deterioration. Exploration of T2/FLAIR-derived dynamics as a treatment response predictor, including evaluation of the developed framework, is currently ongoing. By excluding patients whose progression was unrelated to the T1post MRI signal, the model achieves an NPV of 1.0, with a sensitivity of 1.0 and a specificity of 0.75 without false positives. These results motivate prospective evaluation of early tumor volume dynamics in T1post measurements to predict progression.

As demonstrated, model parameters are not uniquely independently identifiable given the clinical data. Additional pre-treatment tumor growth data may help to constrain the model parameter space and help to further develop the model. Herein, we chose parameter pairs based on statistical considerations to maximize model predictive power and reported these values for reproducibility. However, modification of the functional form of the developed model terms or inclusion of additional mechanisms will change each parameter value. Therefore, herein, estimated parameter values should neither be taken as biological ground truth, nor are they translatable to different mathematical models.

## Figures and Tables

**Table 1:** Summary of model parameters.

Parameter	Unit	Meaning	Bounds	Patient-Specific
λ	day^−1^	Net growth rate in the absence of therapy	[0,ln(2)]	
$\gamma_{0}$	day^−1^	Initial treatment sensitivity	[0,1]	
ε	day^−1^	Evolution of resistance rate	[0,0.1]	✓

**Table 2:** Prediction results of various progression criteria. Progression is defined based on predicted T1post tumor volume relative to nadir. Each progression criterion is defined by a threshold (x% increase in T1post tumor volume from nadir) above which progression is called. The optimal progression criterion is found to be 200% based on maximizing the negative predictive value (NPV). We also report the average number of false negatives (FNs) and how early we predict progression based on the average number of scans and weeks before actual progression.

Progression Criterion	NPV	FN	Mean Scans Predicted Early	Median Weeks Predicted Early	Mean Weeks Predicted Early
0%	0.25	3	3.7	12	18.5
25%	0.57	3	3.3	12	17.2
50%	0.79	3	2.6	10	14
75%	0.79	3	2.6	10	14
100%	0.82	3	2.2	10	12
125%	0.82	3	2.2	10	12
150%	0.83	3	2.1	10	11.7
175%	0.83	3	2.1	10	11.7
200%	0.84	3	2.0	9.3	11.3
225%	0.76	5	2.3	10	13.2
250%	0.76	5	2.3	10	13.2
275%	0.81	5	1.6	9.3	8.1
300%	0.81	5	1.6	9.3	8.1

## Supplementary Materials

Supplementary materials can be found at https://www.mdpi.com/2077-0383/9/7/2019/s1.

## References

[1] Wainwright D.A., Chang A.L., Dey M., Balyasnikova I.V., Kim C.K., Tobias A., Cheng Y., Kim J.W., Qiao J., Zhang L. (2014). Durable therapeutic efficacy utilizing combinatorial blockade against IDO, CTLA-4, and PD-L1 in mice with brain tumors. Clin. Cancer Res..

[2] Berghoff A.S., Kiesel B., Widhalm G., Rajky O., Ricken G., Woehrer A., Dieckmann K., Filipits M., Zielinski C., Marosi C. (2014). PD1 and PD-L1 expression in glioblastoma. J. Clin. Oncol..

[3] Berghoff A.S., Kiesel B., Widhalm G., Rajky O., Ricken G., Woehrer A., Dieckmann K., Filipits M., Brandstetter A., Weller M. (2015). Programmed death ligand 1 expression and tumor-infiltrating lymphocytes in glioblastoma. Neuro Oncol..

[4] Yao Y., Tao R., Wang X., Wang Y., Mao Y., Zhou L.F. (2009). B7-H1 is correlated with malignancy-grade gliomas but is not expressed exclusively on tumor stem-like cells. Neuro Oncol..

[5] Friedman H.S., Prados M.D., Wen P.Y., Mikkelsen T., Schiff D., Abrey L., Yung W.K., Paleologos N., Nicholas M.K., Jensen R. (2009). Bevacizumab Alone and in Combination With Irinotecan in Recurrent Glioblastoma. J. Clin. Oncol..

[6] Kreisl T.N., Kim L., Moore K., Duic P., Royce C., Stroud I., Garren N., Mackey M., Butman J., Camphausen K. (2009). Phase II trial of single-agent bevacizumab followed by bevacizumab plus irinotecan at tumor progression in recurrent glioblastoma. J. Clin. Oncol..

[7] McGranahan T., Therkelsen K.E., Ahmad S., Nagpal S. (2019). Current State of Immunotherapy for Treatment of Glioblastoma. Curr. Treat. Options Oncol..

[8] Reardon D.A., De Groot J.F., Colman H., Jordan J.T., Daras M., Clarke J.L., Nghiemphu P.L., Gaffey S.C., Peters K.B. (2016). Safety of pembrolizumab in combination with bevacizumab in recurrent glioblastoma (rGBM). J. Clin. Oncol..

[9] Sahebjam S., Forsyth P., Arrington J., Jaglal M., Tran N.D., Etame A.B., Wicklund M., Drury-Sibiga A., Long W., Evernden B. (2017). ATIM-18. A phase I trial of hypofractionated stereotactic irrradiation (HFSRT) with pembrolizumab and bevacizumab in patients with recurrent high grade glioma (NCT02313272). Neuro-Oncology.

[10] Wen P.Y., Macdonald D.R., Reardon D.A., Cloughesy T.F., Sorensen A.G., Galanis E., DeGroot J., Wick W., Gilbert M.R., Lassman A.B. (2010). Updated response assessment criteria for high-grade gliomas: Response assessment in Neuro-oncology working group. J. Clin. Oncol..

[11] Ellingson B.M., Cloughesy T.F., Lai A., Nghiemphu P.L., Mischel P.S., Pope W.B. (2011). Quantitative volumetric analysis of conventional MRI response in recurrent glioblastoma treated with bevacizumab. Neuro Oncol..

[12] Gahrmann R., Bent M.J.V.D., Van Der Holt B., Vernhout R.M., Taal W., Vos M., De Groot J.C., Beerepoot L.V., Buter J., Flach Z.H. (2017). Comparison of 2D (RANO) and volumetric methods for assessment of recurrent glioblastoma treated with bevacizumab-a report from the BELOB trial. Neuro Oncol..

[13] Huang R. (2017). Response assessment in high-grade glioma: Tumor volume as endpoint. Neuro Oncol..

[14] Brady R., Enderling H. (2019). Mathematical Models of Cancer: When to Predict Novel Therapies, and When Not to. Bull. Math. Biol..

[15] Claret L., Gupta M., Han K., Joshi A., Sarapa N., He J., Powell B., Bruno R. (2013). Evaluation of Tumor-Size Response Metrics to Predict Overall Survival in Western and Chinese Patients With First-Line Metastatic Colorectal Cancer. J. Clin. Oncol..

[16] Alfonso J.C., Köhn-Luque A., Stylianopoulos T., Feuerhake F., Deutsch A., Hatzikirou H. (2016). Why one-size-fits-all vaso-modulatory interventions fail to control glioma invasion: In silico insights. Sci. Rep..

[17] Baldock A.L., Ahn S., Rockne R.C., Johnston S., Neal M.L., Corwin D., Clark-Swanson K., Sterin G., Trister A.D., Malone H. (2014). Patient-Specific Metrics of Invasiveness Reveal Significant Prognostic Benefit of Resection in a Predictable Subset of Gliomas. PLoS ONE.

[18] Baldock A.L., Yagle K., Born N.E., Ahn S., Trister A., Neal M.L., Johnston S.K., Bridge C.A., Basanta D., Scott J. (2014). Invasion and proliferation kinetics in enhancing gliomas predict IDH1 mutation status. Neuro Oncol..

[19] Harpold H.L., Alvord E.C., Swanson K.R. (2007). The evolution of mathematical modeling of glioma proliferation and invasion. J. Neuropathol. Exp. Neurol..

[20] Jackson P.R., Juliano J., Hawkins-Daarud A., Rockne R.C., Swanson K.R. (2015). Patient-specific mathematical Neuro-oncology: Using a simple proliferation and invasion tumor model to inform clinical practice. Bull. Math. Biol..

[21] Rockne R.C., Rockhill J.K., Mrugala M., Spence A.M., Kalet I., Hendrickson K., Lai A., Cloughesy T., Alvord E.C., Swanson K.R. (2010). Predicting the efficacy of radiotherapy in individual glioblastoma patients in vivo: A mathematical modeling approach. Phys. Med. Biol..

[22] Swanson K.R., Alvord E.C., Murray J.D. (2003). Virtual resection of gliomas: Effect of extent of resection on recurrence. Math. Comp. Model..

[23] Swanson K.R., Rockne R.C., Claridge J., Chaplain M.A., Alvord E.C., Anderson A.R. (2011). Quantifying the role of angiogenesis in malignant progression of gliomas: In silico modeling integrates imaging and histology. Cancer Res..

[24] Tracqui P., Cruywagen G.C., Woodward D.E., Bartoo G.T., Murray J.D., Alvord E.C. (1995). A mathematical model of glioma growth: The effect of chemotherapy on spatio-temporal growth. Cell Prolif..

[25] Banks H., Tran H. (2009). Mathematical and Experimental Modeling of Physical and Biological Processes.

[26] Chowell G. (2017). Fitting dynamic models to epidemic outbreaks with quantified uncertainty: A primer for parameter uncertainty, identifiability, and forecasts. Infect. Dis. Model..

[27] Miao H., Xia X., Perelson A.S., Wu H. (2011). On Identifiability of Nonlinear ODE Models and Applications in Viral Dynamics. SIAM Rev..

[28] Törnqvist L., Vartia P., Vartia Y.O. (1985). How should relative changes be measured?. Am. Stat..

