# Supplementary material for: Tumor Volume Dynamics as an Early Biomarker for Patient-Specific Evolution of Resistance and Progression in Recurrent High-Grade Glioma
Source: J Clin Med. 2020 Jun 27;9(7):2019. doi: 10.3390/jcm9072019 (PMC7409184; doi:10.3390/jcm9072019)
Supplement: Supplementary file 1 [file jcm-09-02019-s001.pdf]

Supplemental Information

Tumor volume dynamics as an early biomarker for patient-specific evolution of resistance and progression in recurrent high-grade glioma

Daniel J. Glazar, G. Daniel Grass, John A. Arrington, Peter A. Forsyth, Natarajan Raghunand, Hsiang-Hsuan Michael Yu, Solmaz Sahebjam, Heiko Enderling

1. Sensitivity analysis.

In order to determine which parameters may be uniform across the patient population and which parameters need to be patient-specific to adequately describe and predict progression, we perform a sensitivity analysis. [25] We calculate the sensitivity matrix $S = \left[\frac{\partial V}{\partial \lambda} \ \frac{\partial V}{\partial \gamma_0} \ \frac{\partial V}{\partial \varepsilon}\right]$ evaluated at each time an MRI was taken. We then take the 2-norm of each column vector, thus estimating absolute sensitivity across all time. We do this across 20 replicates and average their results, normalizing according to the maximum sensitivity. As a result, we find model output tumor volume to be most sensitive to rate of evolution of resistance ε (Figure S1a). Tumor volume was found to be relatively insensitive to net growth rate λ and initial treatment sensitivity γ_0. Therefore, we keep ε to be patient-specific, and make λ and γ_0 to be uniform across all patients. An example of time-dependent sensitivities is shown in Figure S1b for a representative patient across continuous time. Notice that the magnitude of model sensitivity to ε exceeds those sensitivities to λ and γ_0 and each point in time. Also notice given that we fix the model solution to the final observation, sensitivity is 0 at that point.

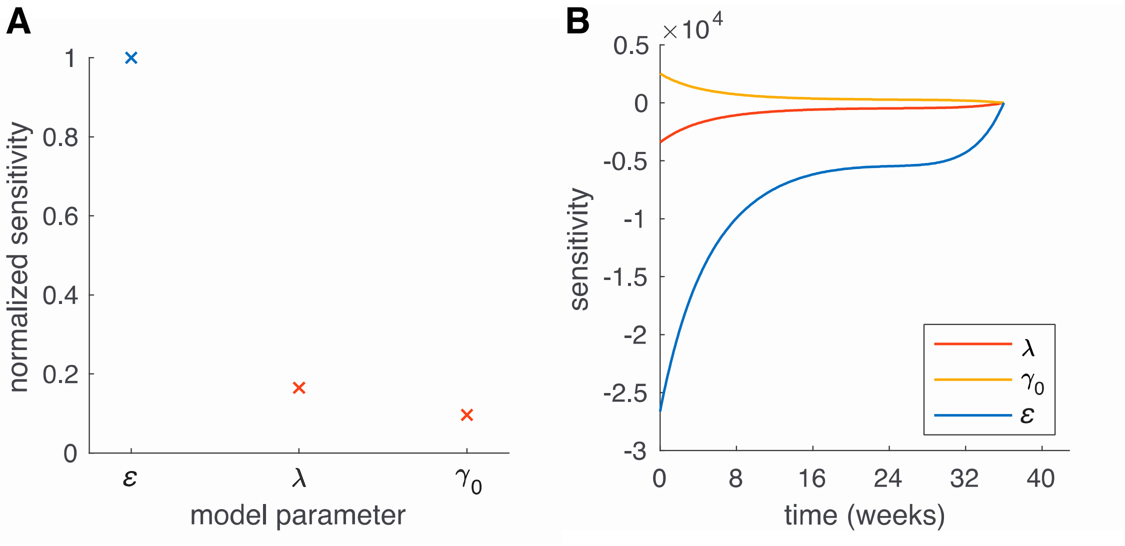

Figure S1. Sensitivity analysis. (**a**) Model output tumor volume is most sensitive to rate of evolution of resistance ε. Therefore, we keep ε to be patient-specific and make λ and γ_0 to be uniform across all patients. (**b**) Time-dependent sensitivities of tumor volume to model parameters for representative patient.

2. Identifiability analysis.

In order to ensure that model parameter values are indeed estimable, we perform an identifiability analysis. [26-27]

2.1. Structural identifiability

In this section, we prove that the base tumor growth and inhibition (TGI) model is indeed identifiable. This is a pre-requisite to further practical non-identifiability analysis. The model is practically identifiable only if it is structurally identifiable.

Claim: The TGI model is structurally identifiable.
Proof: We need to show that $\forall t \in \mathbb{R}, V(t, \bar{\theta}_1) = V(t, \bar{\theta}_2) \Rightarrow \bar{\theta}_1 = \bar{\theta}_2$.
Let $\bar{\theta}_1 = [\lambda_1 \;\; \gamma_{0,1} \;\; \varepsilon_1]$, $\bar{\theta}_2 = [\lambda_2 \;\; \gamma_{0,2} \;\; \varepsilon_2]$, such that $V(t, \bar{\theta}_1) = V(t, \bar{\theta}_2) \;\; \forall t \in \mathbb{R}$.
Define $f(t) := \dot{V}(t, \bar{\theta}_1) - \dot{V}(t, \bar{\theta}_2)$
$= \lambda_1 - \lambda_2 - \gamma_{0,1} \cdot e^{-\varepsilon_1 \cdot t} + \gamma_{0,2} \cdot e^{-\varepsilon_2 \cdot t}$
$= 0.$
Then $\forall n \in \mathbb{Z}$, $f^{(n)}(t) = (-1)^n \cdot \gamma_{0,1} \cdot \varepsilon_1^n \cdot e^{-\varepsilon_1 \cdot t} + (-1)^{n-1} \cdot \gamma_{0,2} \cdot \varepsilon_2^n \cdot e^{-\varepsilon_2 \cdot t}$
$= 0.$
So $\gamma_{0,1} = \frac{\gamma_{2,0} \cdot \varepsilon_2 \cdot e^{(\varepsilon_1 - \varepsilon_2)t}}{\varepsilon_1^n}$
In particular, $\gamma_{0,1} = \frac{\gamma_{0,2} \cdot \varepsilon_2 \cdot e^{(\varepsilon_1 - \varepsilon_2)t}}{\varepsilon_1} = \frac{\gamma_{2,0} \cdot \varepsilon_2^2 \cdot e^{(\varepsilon_1 - \varepsilon_2)t}}{\varepsilon_1^2}$.
Therefore, $\varepsilon_1 = \varepsilon_2$, which implies $\gamma_{1,0} = \gamma_{2,0}$ and $\lambda_1 = \lambda_2$.
Ergo, $\theta_1 = \theta_2$, and the TGI model is structurally identifiable. ■

2.2. Practical identifiability

To determine practical (non-)identifiability, we estimate parameters for the final, reduced model with uniform net growth rate λ and initial treatment sensitivity γ_0 across 20 replicates. We plot the results below in Figure S2. The estimated uniform model parameters are highly correlated with Pearson correlation coefficient $\rho = 1.00$, making the reduced model practically non-identifiable. We therefore set the least sensitive parameter γ_0 to a nominal value that maximizes R^2 ($\gamma_0 = 0.4608$ day^{-1}, $R^2 = 0.78$).

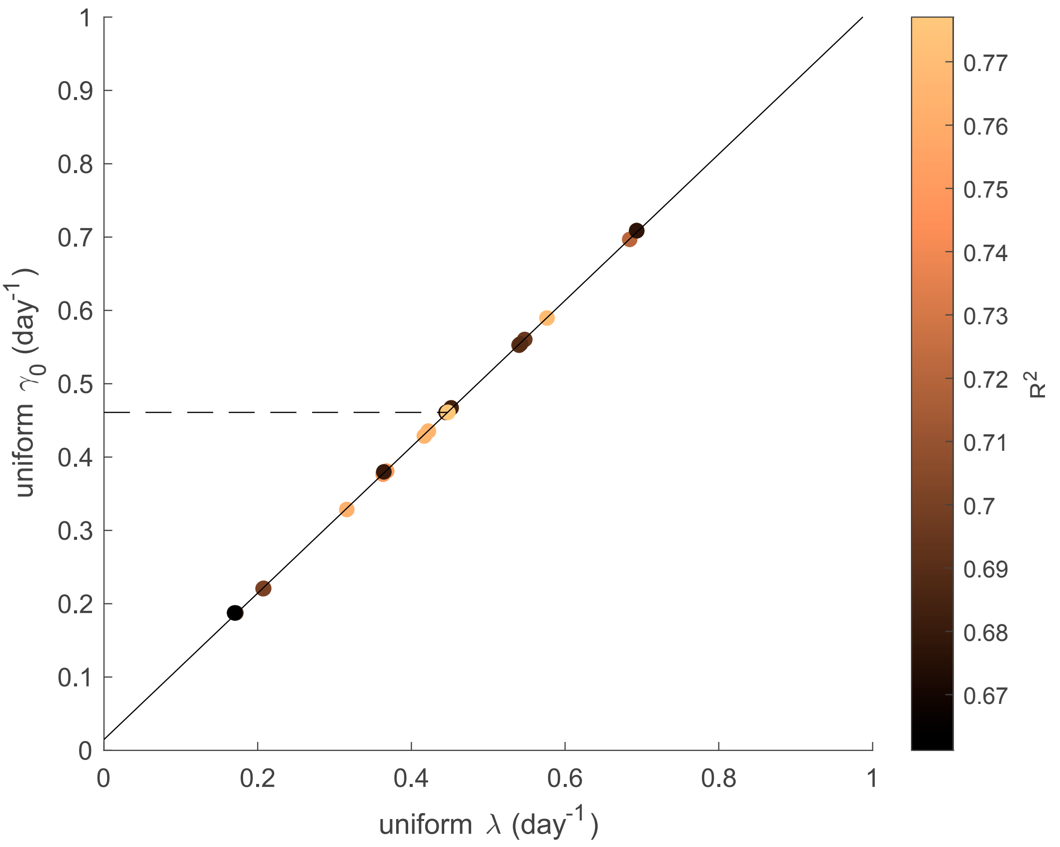

Figure S2. Model is practically non-identifiable. Uniform model parameters are highly correlated (Pearson correlation coefficient $\rho = 1.00$), and the model is practically non-identifiable. We set the least sensitive parameter γ_0 to a nominal value that maximizes R^2 ($\gamma_0 = 0.4608$ day^{-1}, $R^2 = 0.78$).
